# Dynamic handgrip exercise for the detection of myocardial ischemia using fast Strain-ENCoded cardiovascular magnetic resonance

**DOI:** 10.1016/j.jocmr.2025.101879

**Published:** 2025-03-12

**Authors:** Andreas Ochs, Michael Nippes, Janek Salatzki, Lukas D. Weberling, Nael Osman, Johannes Riffel, Hugo A. Katus, Matthias G. Friedrich, Norbert Frey, Marco M. Ochs, Florian André

**Affiliations:** a Department of Cardiology, University of Heidelberg, Heidelberg, Germany; b DZHK (German Centre for Cardiovascular Research), Heidelberg, Germany; c Department of Radiology and Radiological Science, School of Medicine, John Hopkins University, Baltimore, Maryland, USA; d Myocardial Solutions, Inc, Morrisville, North Carolina, USA; e Department of Cardiology, Robert-Bosch-Hospital, Stuttgart, Germany; f Departments of Medicine and Diagnostic Radiology, McGill University Health Centre, Montreal, Quebec, Canada; g Department of Cardiology, University Hospital Frankfurt, Frankfurt am Main, Germany

**Keywords:** FSENC, Handgrip, Longitudinal strain, Ischemia, CMR

## Abstract

**Background:**

Previous data suggest dynamic handgrip exercise (DHE) as a potential physiological, needle-free stressor feasible for cardiovascular magnetic resonance (CMR) examinations. DHE-fast Strain-ENCoded imaging (fSENC) is potentially cost-saving, ultra-fast and avoids pharmacological side effects thereby targeting the drawbacks of conventional pharmacological stress CMR.

**Objectives:**

To assess the diagnostic accuracy of DHE-fSENC for detecting ischemia-related wall motion abnormalities in suspected obstructive coronary artery disease (CAD).

**Methods:**

Patients with known or suspected obstructive CAD referred for CMR stress testing were prospectively enrolled. Diagnostic accuracy was assessed in comparison to pharmacological stress CMR and in a subgroup, compared to invasive coronary angiography (ICA). The CMR protocol was extended by both-handed DHE with 80 repetitions per minute over 2 min followed by fSENC short-axis acquisition before pharmacological stress testing. Stress-induced impairment of regional longitudinal strain was graded suspicious for obstructive CAD.

**Results:**

Two-hundred sixty individuals with cardiovascular high-risk profile (64 ± 13 years, 75% male) were enrolled. DHE-fSENC provided a sensitivity of 79% (95% CI: 64–89) and specificity of 87% (95% CI 82–91) compared to pharmacological stress CMR. In a subgroup of 105 patients with recent ICA, high diagnostic accuracy was found for the detection of obstructive CAD (sensitivity 82% [95% CI: 67–92], specificity 89% [95% CI: 78–95]). Exam duration of DHE-fSENC was significantly reduced compared to conventional CMR stress protocols (DHE-fSENC 207 ± 69 s vs. adenosine-perfusion 287±82 s vs. dobutamine-cine 1132±294 s, all p<0.001).

**Conclusion:**

DHE-fSENC allows for a reliable and fast detection of obstructive CAD, thereby expanding the applicability of needle-free CMR stress testing.

## Graphical abstract

**Figure:**
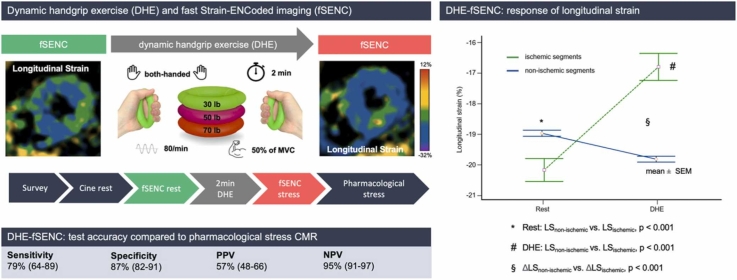
DHE and fSENC imaging. Schematic visualization of the both-sided, DHE at approx. Fifty percentage of MVC and a frequency of 80/min including fSENC acquisition at rest and during DHE. The comparison between fSENC at rest and after DHE of the apical slice in this example shows an impairment of LS after DHE of the inferior apical wall. DHE and fSENC acquisition was integrated into CMR routine protocol and was performed before pharmacological stress. DHE-fSENC: response of LS. Comparison of LS at rest and during DHE. In ischemic segments, LS was significantly impaired during DHE. In contrast, LS was more pronounced during DHE in non-ischemic segments. DHE-fSENC: test accuracy compared to pharmacological stress CMR. Test accuracy of DHE-fSENC compared to pharmacological stress CMR was excellent with a sensitivity of 79% (64–89) and a specificity of 87% (82–91). *CMR* cardiac magnetic resonance imaging, *DHE* dynamic handgrip exercise, *fSENC* fast Strain-ENCoded imaging, *LS* longitudinal strain, *MVC* maximum voluntary contraction, *NPV* negative predictive value, *PPV* positive predictive value, *SEM* standard error of the mean.

## 1 Introduction

Stress testing by cardiovascular magnetic resonance imaging (CMR) has been shown to be a highly accurate non-invasive method to detect myocardial ischemia due to obstructive coronary artery disease (CAD) and is therefore recommended by current guidelines for guiding therapy in patients with stable chest pain [1], [2], [3], [4], [5].

However, the overall low availability of CMR due to the need for high technical and time expenditure, highly trained personnel as well as the need for stress drugs and contrast agents have limited its clinical use in the past. Exercise CMR represents a drug- and needle-free alternative that may overcome some of these shortcomings.

Recently, our group proposed a modified dynamic handgrip exercise (DHE) as a promising physiological maneuver with dobutamine-equivalent effects on heart rate (HR) and myocardial contractility [6]. In contrast to previously evaluated isometric handgrip exercise, the positive inotropic and chronotropic effects appear to be more pronounced [7], [8], [9], [10]. The cardiovascular impact of DHE is supposed to result in an increase in myocardial oxygen demand, thereby unmasking stress-induced ischemia resulting in regional wall-motion abnormalities. To ensure accurate detection of functional impairment on a segmental level and to fulfill the needs of a fast acquisition, we sought to combine DHE with fast Strain-ENCoded imaging (fSENC) and assess the diagnostic accuracy of this needle-free approach to detect myocardial ischemia [11], [12].

## 2 Methods

### 2.1 Study population and design

Patients referred to our center for CMR stress test with known or suspected obstructive CAD were prospectively enrolled. The CMR exams were part of the clinical routine, the indications varied from patients with prior ICA with stenoses of unclear hemodynamic significance, patients with suspected CAD due to symptoms like chest pain or dyspnea at exertion or a high cardiovascular risk profile as well as suspected progression of already known CAD including in- and outpatients.

**Fig. 1:**
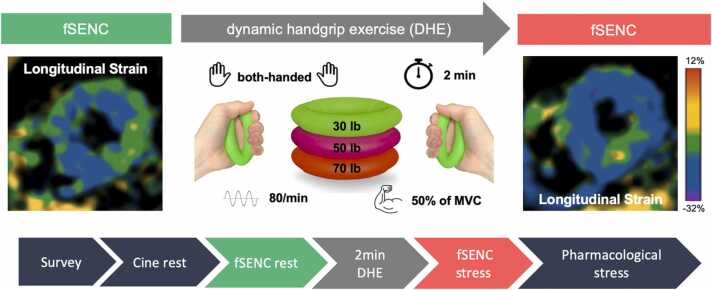
DHE and fSENC imaging. Schematic visualization of the both-sided, DHE at approx. Fifty precentage of MVC and a frequency of 80/min including fSENC acquisition at rest and during DHE. The comparison between fSENC at rest and after DHE of the apical slice in this example shows an impairment of LS after DHE of the inferior apical wall. DHE and fSENC acquisition was integrated into CMR routine protocol and was performed before pharmacological stress. *DHE* dynamic handgrip exercise, *fSENC* fast Strain-ENCoded imaging, *MVC* maximum voluntary contraction, *LS* longitudinal strain

All patients underwent an extended study protocol including DHE as a physiologic stressor prior to pharmacological stress testing (Fig. 1). Adenosine first-pass perfusion was the standard for pharmacological stress testing. Dobutamine was applied in patients with asthma, severe renal failure, prior coronary artery bypass graft surgery (CABG), known severe multi-vessel disease, or large/multiple infarct scars to additionally evaluate myocardial viability. Exclusion criteria included physical impairment with inability to perform DHE, e.g. due to neurological or orthopedic diseases. Patients were asked to complete a dedicated questionnaire regarding symptoms, risk factors, or relevant allergies. Recent invasive coronary angiography (ICA) within 1 year from the index CMR examination was available in a subgroup of patients which was performed before or after CMR.

Other subgroups were defined dependent on a positive or negative stress test as observed in standard pharmacological stress CMR.

The study was approved by the local ethics committee (S-835/2019) and was in accordance with the Declaration of Helsinki. All patients gave written informed consent.

### 2.2 Dynamic handgrip exercise

DHE was performed according to the previously published protocol, involving both-sided, repetitive hand contractions over two minutes (Fig. 1) [6]. CMR-capable rubber handgrip rings in three different strengths were available (30 lb, 50 lb, 70 lb). A dynamic handgrip trainer was used to quantify maximum voluntary contraction (MVC) for each patient. The handgrip ring closest to 50% of MVC was selected to perform handgrip exercise [6], [13].

DHE was performed metronome-guided at a frequency of 80 /min. If necessary, patients could indicate premature exhaustion by pressing the alert bell, triggering immediate initiation of fSENC sequence. The adequate execution of DHE was supervised by the attending technician via visual control and a significant increase of HR, which was continuously monitored by electrocardiogram.

### 2.3 CMR acquisition protocol

CMR was performed on a 1.5T or 3T clinical scanner (Ingenia Cx and Ingenia, Philips Healthcare, Best, The Netherlands). R-wave triggered balanced steady-state free precession (bSSFP) cine images were acquired in three long-axis (2-, 3-, 4-chamber views) and short-axis views covering the whole left ventricle (LV) with 35 phases per cardiac cycle.

**Fig. 2:**
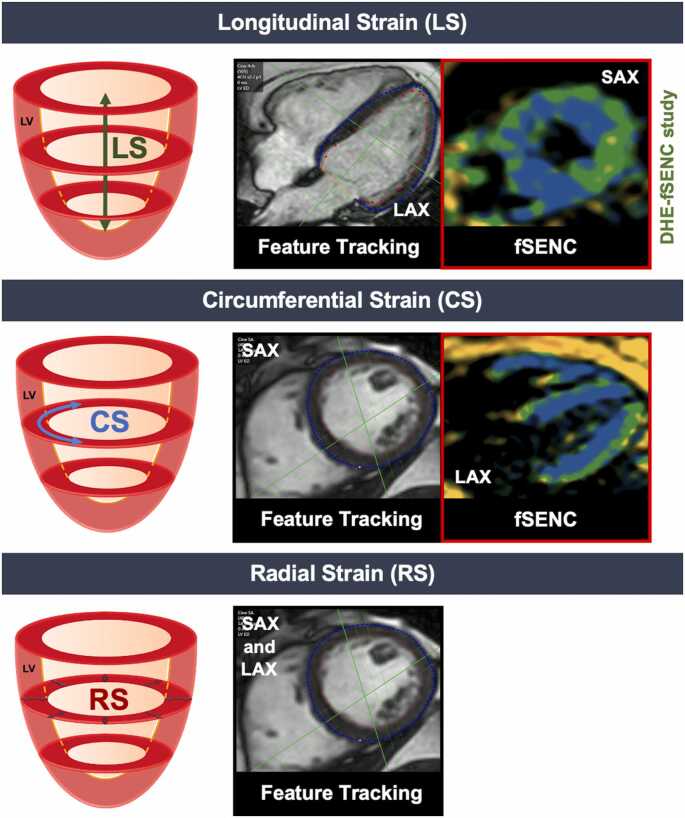
Overview of left ventricular strain directions and their corresponding CMR planes. In contrast to commonly used CMR post-processing tools like feature tracking or speckle-tracking echocardiography, short-axis fSENC slices are required for the assessment of LS, long-axis fSENC slices are required for CS. RS cannot be assessed using fSENC. In this DHE-fSENC study, only LS was assessed. *LS* longitudinal strain, *CS* circumferential strain, *RS* radial strain, *LAX* long axis, *SAX* short axis, *fSENC* fast Strain-ENCoded imaging, *DHE* dynamic handgrip exercise, *CMR* cardiovascular magnetic resonance

The acquisition of the fSENC series was performed as a single heartbeat per slice acquisition, as previously reported [14]. To assess longitudinal strain (LS), short-axis stacks were acquired at the basal, midventricular and apical levels (three slices). As demonstrated in Fig. 2, this stands in opposite to the commonly necessary long-axis views for the assessment of LS using CMR post-processing tools like feature tracking or speckle-tracking echocardiography. The study protocol included fSENC series at rest and immediately after the end of DHE (Fig. 1). DHE-fSENC was performed after the bSSFP cine images at rest and before the pharmacological stress.

As previously described, adenosine stress infusion was started at a rate of 140 µg/kg/min [15]. If no adequate increase of HR or decrease of blood pressure was observed after 2 min, infusion rate was increased to 210 µg/kg/min, however not exceeding a total infusion time of 4 min. Gadolinium-based contrast agent (Gadovist®, Gadobutrol, Bayer, Leverkusen, Germany) was administered intravenously after a HR increase >10/minute or a blood pressure drop of >10 mmHg. Perfusion images were acquired at three levels namely: a basal, midventricular and an apical level.

In the dobutamine stress test, infusion rate started at 10 µg/kg body weight/minute with subsequent increments of 10 µg/kg body weight/minute every 3 min, with a maximum rate of 40 µg/kg body weight/minute. Up to 2 mg of atropine was applied incrementally to achieve the target HR (85% × [220-age]), if necessary. In total, six slices including three short-axis (apical, midventricular and basal) and three long-axis cine images (2-, 3-, 4- chamber view) were acquired at each stress level [15].

### 2.4 Analyses of CMR stress test, fSENC sequences, and coronary angiography

CMR images were analyzed using commercially available workstations (Intellispace Portal V.12, Philips Healthcare, Best, The Netherlands) according to recent guidelines for interpretation and post-processing in CMR [16]. LV volume and ejection fraction measurements including LV mass were assessed using cvi^42^® (v5.13.7, Circle Cardiovascular Imaging, Calgary, Alberta, Canada). Myocardial perfusion images in adenosine stress were only visually analyzed and correlated to LGE if available according to recent recommendations [16].

For the analysis of fSENC series at rest and during DHE including measurements of LS dedicated software (Myostrain® 5.2.3 Myocardial Solutions, Inc., Morrisville, North Carolina) was used. For the detection of myocardial wall motion abnormalities, two independent, trained examiners (A.O., M.N.), who were blinded to the clinical information, compared color-coded fSENC images at rest and during stress. Ischemia was identified as a subendocardial impairment of LS in end-systole of at least one segment in fSENC images during DHE in visual assessment (Fig. 1), as there is currently no established cut-off for ischemia in fSENC. In a second step, segmental LS was quantified by manual contouring of endo- and epicardial borders in end-systole and compared between “positive” and “negative” segments as previously classified.

All analyzed territories in CMR stress test and in fSENC analyses were assigned to different coronaries according to the guidelines of the American Heart Association [17].

ICA was analyzed by experienced interventional cardiologists using Centricity software (GE Medical Systems, Milwaukee, Wisconsin). Obstructive CAD was defined by lumen narrowing ≥75% in left main or proximal left anterior descending artery, as well as >90% in any other vessel according to recent guidelines [3], [18]. In patients with prior ICA, treated stenosis with percutaneous coronary intervention (PCI) were not considered as a significant stenosis in the following CMR.

### 2.5 Reproducibility

To assess intra- and interobserver reproducibility of DHE-fSENC stress test interpretation, 30 randomly selected patients were analyzed a second time. The initial analysis and the repetition for intraobserver reproducibility was conducted at least four weeks apart to minimize the potential for a recall bias. For interobserver reproducibility another examiner repeated analysis for the same 30 patients. The observers were blinded to the results of prior analyses.

### 2.6 Statistical analysis

Normal distribution was assessed using Shapiro-Wilk test. Continuous parameters were expressed as mean ± standard deviation for parametric and as median with interquartile range for nonparametric values. For the comparison of continuous variables between two groups, Student’s t-test and Mann Whitney U test were used as applicable. Not normal distributed continuous variables were tested for differences using the nonparametric Wilcoxon test. The test accuracies of different modalities were compared using the area under the curve (AUC), which was assessed by receiver operating characteristic (ROC) analysis. Cohen's kappa was calculated to check the agreement of the different tests. The intra- and interobserver variability was assessed by the intra-class correlation coefficient with a two-way random model with absolute agreement. Dedicated software (MedCalc statistical software version 22.016, Mariakerke, Belgium) was used for statistical analysis. A p-value of <0.05 was regarded as statistically significant.

## 3 Results

### 3.1 Study population

**Fig. 3:**
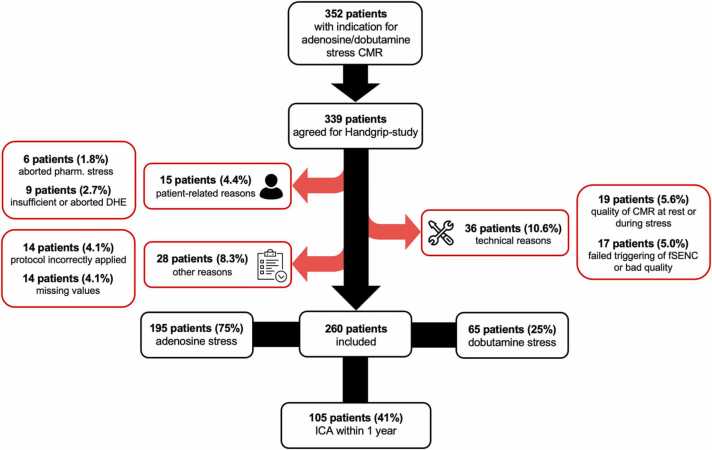
Flowchart of patient recruitment. Three hundred thirty nine patients agreed to the handgrip study. In total, 79 patients had to be excluded mainly because of technical reasons including bad CMR image quality or failed triggering/bad quality of fSENC sequence leading to a final study population of 260 patients. *CMR* cardiac magnetic resonance imaging, *DHE* dynamic handgrip exercise, *fSENC* fast strain-encoded imaging, *ICA* invasive coronary angiography

The final study population consisted of 260 patients (195/260 men, 75%) with an age of 64 ± 13 years (Table 1). Initially, 339 patients agreed to the study and underwent CMR with the study protocol including DHE. However, 79 patients (79/339, 23.3%) had to be excluded (Fig. 3); 36 patients (36/339, 10.6%) due to technical reasons, particularly low quality of CMR images at rest or during stress (19/339, 5.6%) or low fSENC series quality (17/339, 5.0%); 15 patients (15/339, 4.4%) were excluded due to patient-related reasons such as abortion of pharmacological stress (6/339, 1.8%) or the inability to perform the DHE adequately, as well as a prematurely aborted DHE after less than 90 s (9/339, 2.7%); and 28 patients (28/339, 8.3%) because of other reasons such as an incorrect application of the study protocol.

**Table 1:** Demographics table.

	Main group (n = 260)	Pharmacological stress negative patients (n = 213)	Pharmacological stress positive patients (n = 47)	p-value
*Demographics*				
Age, years	64 ± 13	62.9 ± 13.9	68.1 ± 10.0	**0.016**
Male gender, n (%)	195 (75)	156 (73.2)	39 (83.0)	0.164
Weight, kg	82 ± 16	82.3 ± 15.7	81.1 ± 14.6	0.628
Height, cm	174 ± 9	173.8 ± 9.2	173.1 ± 6.2	0.592
BMI, kg/m²	27 ± 5	27.2 ± 4.6	27.1 ± 4.5	0.853
Sinus rhythm, n (%)	238 (91.5)	194 (91.1)	44 (93.6)	0.692
*Cardiovascular risk factors*				
Hypertension, n (%)	180 (69.2)	144 (67.6)	36 (76.6)	0.263
Diabetes, n (%)	52 (20.0)	36 (16.9)	16 (34.0)	**0.011**
Hypercholesterinemia, n (%)	153 (58.8)	116 (54.5)	37 (78.7)	**0.003**
Family history of CAD, n (%)	79 (30.4)	57 (26.8)	22 (46.8)	**0.009**
Smoker, n (%)	104 (40.0)	90 (42.3)	14 (29.8)	0.100
Obesity, n (%)	59 (22.7)	47 (22.1)	12 (25.5)	0.609
*Medical history*				
Known CAD, n (%)	182 (70.0)	140 (65.7)	42 (89.4)	**<0.001**
One-vessel CAD, n (%)	58 (22.3)	48 (22.5)	10 (21.3)	0.889
Two-vessel CAD, n (%)	38 (14.6)	30 (14.1)	8 (17.0)	0.570
Three-vessel CAD, n (%)	86 (33.1)	62 (29.1)	24 (51.1)	**0.007**
Prior PCI, n (%)	102 (39.2)	79 (37.1)	23 (48.9)	0.152
Prior CABG, n (%)	17 (6.5)	14 (6.6)	3 (6.4)	0.955
Prior myocardial infarction, n (%)	72 (27.8)	57 (26.8)	15 (31.9)	0.547
ICA ever performed, n (%)	183 (70.4)	140 (65.7)	43 (91.5	**<0.001**
Recent ICA (<1 year)	105 (40.4)	66 (31.0)	39 (83.0)	**<0.001**
Relevant stenosis ICA, n (%)	53 (29.0)	21 (15.0)	32 (74.4)	**<0.001**
*CMR*				
1.5 Tesla MR scanner, n (%)	134 (51.5)	105 (49.3)	29 (61.7)	0.124
LV ejection fraction, %	58 ± 11	57.5 ± 11.3	59.9 ± 9.1	0.169
LV end-diastolic volume, ml	160 ± 51	162.6 ± 53.3	146.8 ± 36.6	0.055
LV mass, g	115 ± 33	113.5 ± 29.9	119.8 ± 44.2	0.242
LV ejection fraction <50%, n (%)	42 (16.2)	36 (16.9)	6 (12.8)	0.488
LGE performed, n (%)	181 (69.6)	140 (65.7)	41 (87.2)	**0.004**
Any LGE pattern, n (%)	113 (62.4)	81 (57.9)	32 (78.1)	**0.020**
Ischemic LGE, n (%)	66 (36.5)	49 (35.0)	17 (41.5)	0.434
Adenosine stress, n (%)	195 (75.0)	165 (77.5)	30 (63.8)	0.051
Dobutamine stress, n (%)	65 (25.0)	48 (22.5)	17 (36.2)	0.051

Values are ± SD or n (%). p values are for comparison of both subgroups. Significant p-values (p < 0.05) are highlighted in bold. Patient characteristics of the main group and patients with negative or positive pharmacological stress CMR.

*BMI* body mass index, *CABG* coronary artery bypass graft, *CAD* coronary artery disease, *CMR* cardiovascular magnetic resonance imaging, *ICA* invasive coronary angiography, *LGE* late gadolinium enhancement, *LV* left ventricular, *PCI* percutaneous coronary intervention

The patients exhibited a high cardiovascular risk profile with many having a history of myocardial infarction (72/260, 27.8%), prior PCI (102/260, 39.2%), and CABG (17/260, 6.5%) (Table 1). Most patients had a preserved LV ejection fraction, only 42 patients had a reduced ejection fraction <50% (42/260, 16.2%). A few patients had a concomitant cardiomyopathy (20/260, 8%) such as dilatative or hypertrophic cardiomyopathy or a left bundle branch block (16/260, 6.2%).

In 47 patients, of whom 30 underwent adenosine stress perfusion and 17 dobutamine stress, the pharmacological stress test was positive (47/260, 18.1%). The median number of ischemic segments was 2 (range: 1–4) for adenosine and 2 (range: 1–3) for dobutamine, with only 1 ischemic segment in 16 patients (16/260, 6.2%). Patients with a positive stress CMR were significantly older (68.1 ± 10.0 years vs. 62.9 ± 13.9 years, p<0.05) and had more cardiovascular risk factors as well as a more severe CAD (Table 1). No significant differences were found in regard to global longitudinal strain (GLS) at rest or during DHE (Table 2).

**Table 2:** DHE-fSENC parameters of the main group and patients with negative or positive pharmacological stress CMR.

	Main group (n = 260)	Pharmacological stress negative patients (n = 213)	Pharmacological stress positive patients (n = 47)	p-value
DHE completed, n (%)	227 (87.3)	190 (89.2)	37 (78.7)	0.051
Handgrip 30 lb, n (%)	221 (85.0)	180 (84.5)	41 (87.2)	0.637
Handgrip 50 lb, n (%)	38 (14.6)	33 (15.5)	5 (10.6)	0.396
Handgrip 70 lb, n (%)	1 (0.4)	0 (0.0)	1 (2.1)	**0.033**
HR_rest_, /min	68 ± 11	68.8 ± 11.0	66.4 ± 13.2	0.199
HR_DHE_, /min	88 ± 14	88.5 ± 13.5	88.1 ± 14.1	0.833
ΔHR, /min	20 ± 10	19.8 ± 9.7	21.7 ± 8.6	0.212
GLS_rest_,%	−18.5 ± 2.6	−18.6 ± 2.6	−18.2 ± 2.3	0.474
GLS_DHE_, %	−19.3 ± 2.8	−19.4 ± 2.8	−18.7 ± 2.6	0.115
ΔGLS, %	- 0.8 ± 1.5	−0.9 ± 1.6	−0.5 ± 1.3	0.092

Values are ± SD or n (%). p values are for comparison of both subgroups. Significant p-values (p < 0.05) are highlighted in bold.

*CMR* cardiac magnetic resonance imaging, *DHE* dynamic handgrip exercise, *fSENC* fast Strain-ENCoded imaging, *GLS* global longitudinal strain, *HR* heart rate, *lb* pound

### 3.2 Invasive coronary angiography

One hundred five patients (105/260, 40.4%) had undergone an ICA within 1 year from the index CMR, at median 52 (3–131) days before or after CMR. Eighty-one patients (81/105, 77.1%) underwent ICA before CMR—patients who recently had myocardial infarction (22/105, 27.2%) with treatment of the culprit lesion but residual stenoses, patients with single or multiple stenoses of unclear hemodynamic relevance including chronic total occlusion of one coronary vessel (14/105, 17.3%) or left main stem stenosis (11/105, 13.5%) to determine the ischemic burden and to help to plan the optimal revascularization strategy. Twenty-four patients underwent ICA after CMR (24/105, 22.9%), of which 19 patients had prior positive pharmacological stress CMR (19/24, 79.2%), the other patients had the finding of unknown subendocardial LGE (2/24, 8.3%) or a cardiomyopathy (1/24, 4.2%) and two patients had acute myocardial infarction short time after CMR (2/24, 8.3%).

In 43 of these 105 patients (43/105, 41.0%), ICA revealed significant coronary stenoses as defined above. At the vessel level, 70 of 315 coronary vessels showed significant stenosis. Twenty-three patients had significant stenoses in a single vessel, 13 patients had significant stenoses in 2 coronary vessels, and 7 patients had significant stenoses in all 3 coronary vessels.

### 3.3 Dynamic handgrip exercise

In the final study cohort of 260 patients, 33 patients terminated DHE between 90 s and less than 120 s (33/260, 12.7%); yet they were still included in our analysis since fSENC sequence was promptly initiated and a favorable hemodynamic response was observed. Despite the overall quite heterogenous study population with a high burden of cardiovascular disease, the exclusion rate of DHE was less than 10% as mentioned above.

**Fig. 4:**
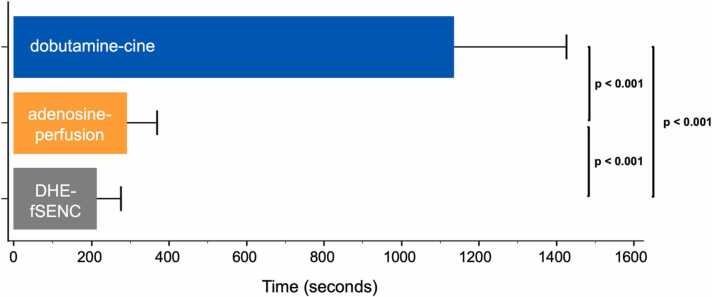
Stress test duration of DHE-fSENC. The duration of DHE-fSENC was significantly reduced compared to standard pharmacological stress tests (p<0.001). *DHE* dynamic handgrip exercise, *fSENC* fast Strain-ENCoded imaging

The duration of DHE-fSENC, including all preparations and the image acquisition during stress (207 ± 69 s), was significantly shorter than of adenosine-perfusion (287 ± 82 s) and dobutamine stress CMR (1132 ± 294 s) (all p<0.001) (Fig. 4).

In patients with suspected or known CAD, significant increase of HR (HR_rest_ vs. HR_DHE_: 68 ± 11/min vs. 88 ± 14/min, p<0.001; ΔHR: 20 ± 10/min, range: 0–55/min) as well as GLS (−18.5 ± 2.6% vs. −19.3 ± 2.8%, p<0.001) was observed during DHE. This held true regardless of a positive or negative pharmacological stress CMR (Table 2). Even in subgroups of patients with betablocker therapy (ΔHR_betablocker_ vs. ΔHR_nobetablocker_: 17 ± 8/min vs. 23 ± 10/min), elderly patients above 80 years (ΔHR_>80years_ vs. ΔHR_<80years_: 19 ± 11/min vs. 20 ± 9/min) and patients with a three-vessel CAD (ΔHR_3vesselCAD_ vs. ΔHR_no3vesselCAD_: 21 ± 10/min vs. 20 ± 10/min) significant increases of HR were observed (HR_rest_ vs. HR_DHE_, p<0.001 for all subgroups).

**Fig. 5:**
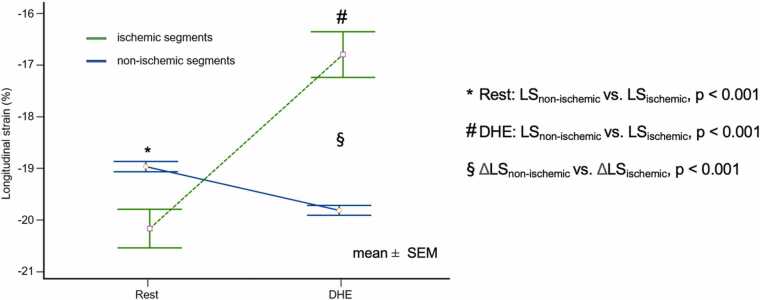
(B) DHE-fSENC: longitudinal strain response. Comparison of LS at rest and during DHE. In ischemic segments, longitudinal strain was significantly impaired during DHE. In contrast, LS was more pronounced during DHE in non-ischemic segments. *DHE* dynamic handgrip exercise, *LS* longitudinal strain, *SEM* standard error of the mean

After visual analysis of fSENC series at rest and during DHE, 139 segments in 65 patients showed subendocardial impairment during DHE suggesting ischemia. Forty-seven patients had suspected ischemia during DHE in 1 corresponding coronary vessel, and 18 patients in multiple corresponding vessels. In ischemic segments detected by DHE-fSENC, an impairment of LS from −20.2 ± 4.4% at rest to −16.8 ± 5.2% (p<0.001) during DHE was observed (Fig. 5). In contrast, in non-ischemic segments LS improved from −19.0 ± 5.0% at rest to −19.8 ± 4.7% during DHE (p<0.001). Ischemic segments exhibited significantly higher LS at rest compared to the non-ischemic segments, during DHE, however, there was a pronounced decrease of LS in ischemic segments (ΔLS_ischemic_: 3.4 ± 3.9%; p<0.001), whilst LS increased in non-ischemic segments (ΔLS_non-ischemic_: −0.8 ± 3.6%; p<0.001). Best differentiation between previously classified “positive” and “negative” segments using ROC curve analysis was observed for ΔLS >+1.2% with an AUC of 0.80 (p<0.001).

Intra- (intra-class correlation coefficient (ICC): 0.96, 95% confidence interval (CI) 0.92–0.98) and interobserver reproducibility (ICC: 0.88, 95% CI 0.75–0.94) for stress test interpretation using DHE-fSENC were excellent.

### 3.4 Stress test accuracy

Compared to the reference of pharmacological stress CMR, DHE-fSENC achieved a good diagnostic accuracy (85% [95% CI: 81–89]) at a patient-level with a sensitivity of 79% (95% CI: 64–89) and a specificity of 87% (95% CI: 82–91) (Table 3). DHE-fSENC correctly detected significant CAD in 37 out of 47 patients and correctly excluded significant CAD in 185 out of 213 patients. The agreement between DHE-fSENC and pharmacological stress CMR was good with a proportion of agreement of 86.5% (k = 0.61 [95% CI: 0.50–0.73]). At a vessel-level, diagnostic accuracy was 83% (95% CI: 79–87), sensitivity was 53% (95% CI: 38–68), the specificity 89% (95% CI: 84–92). At a slice-level, diagnostic accuracy was 87% (95% CI: 84–89), sensitivity 46% (95% CI: 35–56) and the specificity 92% (95% CI: 90–94).

**Table 3:** Stress test accuracy to detect myocardial ischemia or significant obstructive CAD.

	Accuracy	Sensitivity	Specificity	PPV	NPV
*Per-patient level, reference to pharmacological stress CMR (n=260)*					
DHE-fSENC	85 (81–89)	79 (64–89)	87 (82–91)	57 (48–66)	95 (91–97)
*Per-vessel level, reference to pharmacological stress CMR (n=780)*					
DHE-fSENC	83 (79–87)	53 (38–68)	89 (84–92)	45 (35–56)	92 (89–94)
*Per-slice level, reference to pharmacological stress CMR (n=780)*					
DHE-fSENC	87 (84–89)	46 (35–56)	92 (90–94)	43 (34–51)	93 (92–94)
*Per-patient level, reference to ICA (n=105)*					
Pharmacological stress CMR	80 (71–87)	69 (53–82)	87 (76–94)	78 (65–88)	81 (72–87)
Adenosine-Perfusion	79 (68–88)	70 (47–87)	84 (71–94)	70 (52–83)	84 (74–91)
Dobutamine-Cine	81 (64–92)	68 (43–87)	94 (71–100)	93 (65–99)	73 (58–84)
DHE-fSENC	86 (78–92)	82 (67–92)	89 (78–95)	84 (72–91)	87 (79–93)
*Per-vessel level, reference to ICA (n=315)*					
Pharmacological stress CMR	83 (79–87)	46 (34–58)	94 (90–97)	68 (55–79)	86 (83–88)
Adenosine-Perfusion	85 (84–90)	46 (31–63)	94 (89–97)	66 (49–79)	88 (84–90)
Dobutamine-Cine	81 (72–88)	45 (26–64)	94 (86–98)	72 (50–87)	82 (77–87)
DHE-fSENC	84 (79–88)	53 (41–65)	93 (89–96)	67 (56–77)	87 (84–90)

Values are mean (95% confidence intervals).

*CAD* coronary artery disease*, CMR* cardiovascular magnetic resonance, *DHE* dynamic handgrip exercise, *fSENC* fast Strain-ENCoded imaging, *ICA* invasive coronary angiography, *NPV* negative predictive value, *PPV* positive predictive value

In the subgroup of patients with a recent ICA (n = 105), pharmacological stress CMR reached a sensitivity of 69% (95% CI: 53–82) and a specificity of 87% (95% CI: 76–94) to detect significant obstructive CAD on a patient-level (Table 3). In comparison, detection of obstructive CAD using DHE-fSENC had a sensitivity of 82% (95% CI: 67–92) and a specificity of 89% (95% CI: 78–95) (AUC_phStress_ 0,77 vs. AUC_DHE_ 0,84, p=0.117). Thereby, DHE-fSENC correctly detected significant CAD in 35 out of 43 patients and it correctly excluded significant CAD in 55 out of 62 patients. On a vessel-level (reference: ICA), lower test accuracy was shown with a sensitivity of 46% (95% CI: 34–58) and specificity of 94% (95% CI: 90–97) for pharmacological stress CMR and a sensitivity of 53% (95% CI: 41–65) and specificity of 93% (95% CI: 89–96) for DHE-fSENC (AUC_phStress_ 0,70 vs. AUC_DHE_ 0,73, p = 0.442).

Diagnostic accuracy for the detection of myocardial ischemia was tested for different subgroups on a patient-level with pharmacological stress CMR as the reference (Table 4). In a subgroup of patients without known CAD (n = 78), DHE-fSENC had a sensitivity of 100% (95% CI: 48–100) and a specificity of 89% (95% CI: 78–95). Even in populations with high burden of cardiovascular disease, DHE-fSENC achieved an excellent test accuracy, e.g. in patients after CABG (n = 17, sensitivity: 100% [95% CI: 29–100], specificity: 78% [95% CI: 40–97]) or 3-vessel-disease (n = 86, sensitivity: 91% [95% CI: 72–99], specificity: 81% [95% CI: 69–91]). In patients with reduced LV ejection fraction <50%, test accuracy was overall lower but still good (n = 42, sensitivity: 83% [95% CI: 36–100], specificity: 92% [95% CI: 78–98]). In a subgroup of patients with subendocardial, infarct-related LGE, DHE-fSENC test accuracy was also lower compared to the main group (n = 66, sensitivity: 76% [95% CI: 50–93], specificity: 86% [95% CI: 73–94]).

**Table 4:** Stress test accuracy of selected subgroups on per-patient level with reference to pharmacological stress CMR.

	Accuracy	Sensitivity	Specificity	PPV	NPV
*Patients without known CAD (n=78)*					
DHE-fSENC	90 (80–96)	100 (48–100)	89 (78–95)	42 (15–72)	100 (94–100)
*Patients with three-vessel-disease (n=86)*					
DHE-fSENC	84 (74–92)	91 (72–99)	81 (69–91)	68 (49–83)	96 (85–99)
*Patients with reduced LV ejection fraction<50% (n=42)*					
DHE-fSENC	90 (77–97)	83 (36–100)	92 (78–98)	63 (35–84)	97 (85–100)
*Patients after CABG (n=17)*					
DHE-fSENC	83 (52–98)	100 (29–100)	78 (40–97)	60 (31–84)	100 (59–100)
*Patients with subendocardial LGE (n=66)*					
DHE-fSENC	83 (72–91)	76 (50–93)	86 (73–94)	65 (47–79)	91 (82–96)

Values are mean (95% confidence intervals).

*CABG* coronary artery bypass graft, *CAD* coronary artery disease, *CMR* cardiovascular magnetic resonance, *DHE* dynamic handgrip exercise, *fSENC* fast Strain-ENCoded imaging, *LV* left ventricular, *NPV* negative predictive value, *PPV* positive predictive value

**Fig. 6:**
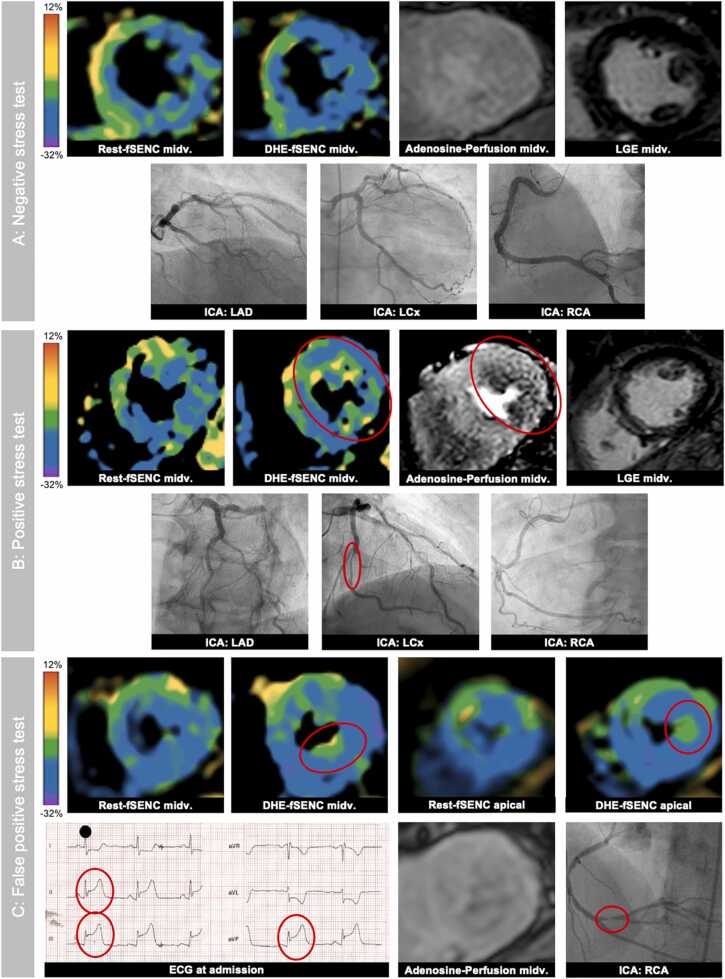
Examples. (A) Negative stress test. 61-year-old, male patient. Progressive dyspnea without typical angina. ICA revealed proximal LAD stenosis with 50% lumen narrowing. Adenosine-perfusion was rated negative. During DHE, the HR increased from 60/min to 98/min, GLS was more pronounced after DHE, from −16.7% to −20.2%. DHE-fSENC was rated negative for ischemia. (B) Positive stress test. 75-year-old, male patient. No dyspnea or typical angina. Former resuscitation during non-ST-elevation myocardial infarction due to LAD stenosis 6 months before. Residual stenosis of LCx (75%). Adenosine-perfusion was rated positive lateral midventricular (2 segments). During DHE HR increased from 54/min to 83/min, GLS slightly increased from −19.0% to −19.5%. In the qualitative analysis of DHE-fSENC a worsening of LS lateral midventricular was observed, similar to adenosine-perfusion – DHE-fSENC was rated positive. (C) “False-positive” stress test. 57-years-old, female patient. Three months before - after progressive, typical angina –PCI including the insertion of a drug-eluting stent into the distal RCA was performed. The patient was referred to stress CMR for residual LCx and LAD stenoses (both 50–75% lumen narrowing). Adenosine-perfusion revealed no significant perfusion deficit. During DHE (HR increase from 75/min to 115/min, GLS increase from −19.6% to −22.5%), a new subendocardial worsening of LS of the inferior wall (2 segments) was detected. Two weeks after CMR the patient presented at chest pain unit with typical angina and ST-segments elevation (II, III, and aVF). In ICA, a subtotal occlusion of RCA, independent of the first lesion, was found and a drug-eluting stent was successfully inserted. *CMR* cardiac magnetic resonance imaging, *DHE* dynamic handgrip exercise, *fSENC* fast Strain-ENCoded imaging, *GLS* global longitudinal strain, *HR* heart rate, *ICA* invasive coronary angiography, *LAD* left anterior descending artery, *LCx* left circumflex artery, *NSTEMI* non-ST-elevation infarction, *PCI* percutaneous coronary intervention, *RCA* right coronary artery

Various examples of DHE-fSENC are shown in Fig. 6.

## 4 Discussion

This pilot study evaluated the diagnostic accuracy of physiological stress testing using DHE in combination with fSENC CMR. The key findings are: (I) Feasibility was good even in this heterogeneous, elderly population, including patients with known CAD and numerous comorbidities. (II) DHE-fSENC showed good sensitivity and specificity for detecting myocardial ischemia or significant obstructive CAD compared with pharmacological stress CMR and ICA.

The diagnostic and prognostic value of pharmacological stress CMR has been demonstrated several times [1]. To minimize the risk of adverse events and enhance the cost-effectiveness of stress CMR, efforts have focused on developing stress CMR protocols that eliminate the need for pharmacologic stressors and gadolinium-based contrast agents [19]. Various CMR-capable devices, such as supine cycle- and stepper ergometers have been developed [19]. However, these have not been widely implemented in clinical practice due to high personnel and technical requirements and often reduced image quality due to motion artifacts.

In contrast, handgrip exercise can be easily performed using commercially available CMR-compatible rubber handgrip rings, despite the lower exercise intensity. Isometric handgrip exercise has previously been shown to induce myocardial ischemia. Early invasive studies observed a significant decrease in myocardial perfusion during handgrip exercise in significant CAD [20], [21]. This finding has been confirmed by non-invasive studies using nuclear magnetic resonance spectroscopy and CMR flow measurements [22], [23]. Therefore, myocardial ischemia results in a dysfunction of subendocardial, predominantly longitudinal myocardial fibers, which can be observed as an impairment of LS [24], [25], [26], [27]. Data on the response of LS after handgrip exercise in patients with obstructive CAD are limited; after isometric handgrip exercise, Ryo et al. observed, using speckle-tracking echocardiography, a more pronounced LS in myocardium corresponding to stenotic coronary vessels and an impairment of LS in myocardium corresponding to non-stenotic coronaries [9]. Our group has previously examined the response of LS to DHE in CAD patients without significant obstruction, finding that DHE induced a high chronotropic and inotropic response in both healthy subjects and CAD patients comparable to an “intermediate” dobutamine stress [6], [28], [29]. Thereby, we considered the effect of DHE to be too small to result in visible wall motion abnormalities in standard cine series. In the current study, we hypothesized that fSENC could detect ischemia or significant obstructive CAD during DHE by observing an impairment of subendocardial LS, as shown previously during hyperventilation/breath-hold maneuver and adenosine stress [30].

CMR-fSENC provides reproducible and rapid assessment of LS. Unlike other strain imaging modalities, such as post-processing tools based on feature tracking, fSENC offers reliable results even at the segmental level - a prerequisite for detecting regional changes in LS after DHE [31]. The segmental strain analysis using fSENC or SENC not only supports and enhances the diagnostic value of CMR in ischemic heart disease and heart failure, but has also been shown to improve the prognostic value and to identify patients at risk for future cardiac events [5], [29], [32], [33], [34]. In contrast, post-processing algorithms based on feature tracking are not dependent on dedicated CMR sequences, they allow for a rapid assessment of LV strain using routinely used cine sequences, particularly global strain values. Feature tracking-based CMR strain studies have also demonstrated additional diagnostic and prognostic value in ischemic heart disease [35], [36]. However, for accurate detection of myocardial ischemia during DHE, a reproducible and fast assessment of segmental and layer-specific subendocardial strain is essential, which is only guaranteed by fSENC [12], [31].

In the subgroup analysis of patients who underwent an ICA, the observed sensitivity of pharmacological stress CMR was lower than literature values, which reported a sensitivity of about 85–90% [4], [37], [38]. In contrast to these studies, we opted for a more heterogenous study population with a high cardiovascular risk profile, including a high percentage of patients with known CAD (77%), prior myocardial infarction (28%), and prior CABG surgery (7%). Furthermore, twenty patients had various cardiomyopathies, which can affect the accuracy of stress perfusion. For example, hypertrophic cardiomyopathy can cause a diffuse subendocardial perfusion deficit, whereas perfusion signals are lower in dilated cardiomyopathy with enlarged ventricles, thin ventricular walls, and low cardiac output. Most previous studies included more selected, healthier populations [4], [38], [39]. Additionally, lower sensitivity for adenosine perfusion has already been shown in patients with atrial fibrillation (sensitivity: 74%), and those after CABG (sensitivity: 77%), which corresponded to 9% and 7% of our study population, respectively [40], [41]. Studies using quantitative ICA as the reference and not fractional flow reserve, reported a similar test accuracy than our study for CMR stress testing [42].

DHE showed good safety and feasibility in a heterogeneous, elderly population, including patients with known CAD and many comorbidities. Only 17 patients had to be excluded due to non-interpretable fSENC series or inadequate electrocardiogram (ECG) triggering due to motion artifacts during DHE. A total of 87.3% of the final study population completed the 120 s of DHE, 16.7% aborted DHE due to peripheral fatigue after 90 to less than 120 s. Only nine patients were excluded due to the abortion of DHE after less than 90 s or an insufficient DHE with a frequency < 80/min.

Providing a good feasibility and diagnostic accuracy including a high negative predictive value, DHE-fSENC may represent a gatekeeper to rule out significant CAD. Combined with highly efficient, contrast agent-free CMR protocols, as previously reported, these exams could be applied to patients with a low risk of cardiac disease [43]. Moreover, if CMR contrast agents are contraindicated, e.g., due to renal failure, DHE-fSENC might be an alternative to dobutamine, which may also be contraindicated in some patients. In case of any pathological finding, needle-free CMR may be expanded with pharmacological stress and late gadolinium enhancement for confirmation and further investigation.

## 5 Limitations

Recent ICA was available in only 105 patients as a reference. The time range between CMR and ICA of up to 12 months is another limitation. Despite the most often very stable course of chronic coronary syndrome, faster progress of stenosis, restenosis, etc. cannot be ruled out and would influence our results. Measurements of the fractional flow reserve, the current reference standard for the invasive hemodynamic evaluation of coronary stenosis, were not available for most patients. The visual assessment of coronary artery stenosis could lead to misinterpretation of the stenosis significance, thus affect the accuracy of stress testing with DHE and pharmacological stress CMR [44].

In comparison to standard pharmacological stress CMR including bSSFP cine or perfusion images, the spatial resolution of fSENC was lower and artifacts were more frequently present. Accordingly, some experience in interpreting fSENC images was required to deal with artifacts correctly and to achieve a good level of accuracy. Additionally, the interpretation of myocardial segments with infarct-related scar was more difficult because LS was already impaired at rest, changes due to DHE were smaller and more difficult to detect. Therefore, DHE-fSENC may not be an optimal approach for a combined assessment of ischemia and viability.

Regarding DHE, another limitation was the lack of dynamic range for the different handgrip strengths we provided: in some patients, even our lowest strength (30 lb) exceeded the intended 50% of MVC. Also, for technical reasons, it was not possible to measure blood pressure during DHE to monitor adequate performance of DHE—neither pulse wave-based finger clips nor classic Riva-Rocci measurements were feasible during hand contractions.

The ischemic burden was low in our population as another limitation of the study; only 47 patients had a positive pharmacological stress CMR, of which 16 patients had only 1 ischemic segment, which in turn means that only 31 patients had significant ischemia of at least 2 segments, as defined by current guidelines [3]. Due to the overall low ischemic burden, the test accuracy of DHE-fSENC could be under- or overestimated.

No quantitative or semi-quantitative analysis of adenosine perfusion imaging was used.

No prognostic data were assessed regarding clinical endpoints or symptoms including symptom relief after PCI as a result of a positive stress test.

A fairly large number of patients (n = 79, 23.3%) had to be excluded from the initial study population, not only but also related to the DHE-fSENC protocol. For a potential clinical use, these study-related exclusions need to be reduced.

## 6 Conclusions

DHE is a promising physiological, needle-free, safe, and rapid maneuver to induce myocardial ischemia, detectable by fSENC-CMR as an impairment of LS. Thereby, DHE-fSENC demonstrates high diagnostic accuracy in identifying significant obstructive CAD. Further multicenter studies using DHE-fSENC are essential to validate the diagnostic and prognostic value in patients with CAD.

## Funding

The study received no external funding.

## Author contributions

**Andreas Ochs:** Conceptualization, Data curation, Formal analysis, Investigation, Methodology, Project administration, Software, Supervision, Validation, Visualization, Writing – original draft, Writing – review & editing. **Michael Nippes:** Data curation, Investigation, Writing – review & editing. **Janek Salatzki:** Data curation, Formal analysis, Investigation, Writing – review & editing. **Lukas D. Weberling:** Data curation, Formal analysis, Investigation, Writing – review & editing. **Nael Osman:** Investigation, Methodology, Software. **Johannes Riffel:** Data curation, Formal analysis, Investigation, Supervision, Writing – review & editing. **Hugo A. Katus:** Resources, Supervision, Writing – review & editing. **Matthias G. Friedrich:** Conceptualization, Methodology, Supervision, Writing – review & editing. **Norbert Frey:** Resources, Supervision, Writing – review & editing. **Marco M. Ochs:** Conceptualization, Data curation, Formal analysis, Investigation, Methodology, Software, Supervision, Validation, Visualization, Writing – original draft, Writing – review & editing. **Florian André:** Conceptualization, Data curation, Formal analysis, Investigation, Methodology, Software, Supervision, Validation, Visualization, Writing – original draft, Writing – review & editing.

## Declaration of Competing Interest

The authors declare the following financial interests/personal relationships which may be considered as potential competing interests: Nael Osman reports a relationship with Myocardial Solutions Inc. that includes employment. Other authors declare that they have no known competing financial interests or personal relationships that could have appeared to influence the work reported in this paper.

## Data Availability

The data that support the findings of this study are available from the corresponding author, AO, upon reasonable request.
